# The Arginine Catabolism-Derived Amino Acid l-ornithine Is a Chemoattractant for *Pseudomonas aeruginosa*

**DOI:** 10.3390/microorganisms10020264

**Published:** 2022-01-24

**Authors:** Basanta Dhodary, Inmaculada Sampedro, Shekooh Behroozian, Victor Borza, Stephanie Her, Jane E. Hill

**Affiliations:** 1 School of Biomedical Engineering, University of British Columbia, Vancouver, BC V6T 1Z4, Canada; basanta02@hotmail.com (B.D.); shekooh@mail.ubc.ca (S.B.); 2 Thayer School of Engineering, Dartmouth College, Hanover, NH 03755, USA; isampedro@ugr.es (I.S.); vaborza@gmail.com (V.B.); stephanie.cindy.her@gmail.com (S.H.); 3 Biomedical Research Center (CIBM), Biotechnology Institute, Avda del Conocimiento s/n, 18100 Armilla, Spain; 4 Department of Microbiology, Faculty of Pharmacy, University of Granada, Campus de Cartuja s/n, 18071 Granada, Spain

**Keywords:** chemotaxis, l-ornithine, *Pseudomonas aeruginosa*, chemoreceptor

## Abstract

*Pseudomonas aeruginosa* is a common, opportunistic bacterial pathogen among patients with cystic fibrosis, asthma, and chronic obstructive pulmonary disease. During the course of these diseases, l-ornithine, a non-proteinogenic amino acid, becomes more abundant. *P. aeruginosa* is chemotactic towards other proteinogenic amino acids. Here, we evaluated the chemotaxis response of *P. aeruginosa* towards l-ornithine. Our results show that l-ornithine serves as a chemoattractant for several strains of *P. aeruginosa*, including clinical isolates, and that the chemoreceptors involved in *P. aeruginosa* PAO1 are PctA and PctB. It seems likely that *P. aeruginosa*’s chemotactic response to l-ornithine might be a common feature and thus could potentially contribute to pathogenesis processes during colonization and infection scenarios.

## 1. Introduction

*Pseudomonas aeruginosa* (*Pa*) is a widely occurring Gram-negative bacterial pathogen which can cause a variety of opportunistic infections in humans worldwide [1,2,3]. For example, *Pa* is the leading cause of nosocomial infections in immunocompromised, cystic fibrosis (CF), cancer and burn patients [1,4,5,6,7,8]. *Pa* is known to be naturally resistant to many antimicrobial agents making its eradication increasingly challenging [9].

The success of a bacterial infection relies, in part, on organism’s management of and adaptation to the host environment. One mechanism utilized by some bacteria is an ability to move towards or away from chemical gradients using their chemosensing and motility apparatuses which may increase their access to desired substances or avoid hostile environments [10,11]. Several studies have revealed that chemotaxis behaviors play an important role in *Pa* infections [12,13]. *Pa* strain PAO1 (PAO1) and by analogy, other strains of *Pa*, has 26 putative methyl-accepting chemotaxis proteins (MCPs) that feed into four chemosensory pathways (Che, Che2, Chp, Wsp) [13]. The MCPs linked to chemosensing of the 20 natural, proteinogenic L-amino acids, are PctA, PctB, and PctC [14,15].

Arginine is one of the most versatile amino acids in human body [16]. Arginine is the precursor to l-ornithine, polyamines, proline, agmatine, and creatinine. Arginine is converted to l-ornithine in the presence of arginase [16,17,18]. Heightened production of arginase in persons with CF, asthma, chronic obstructive pulmonary disease (COPD), pulmonary hypertension and idiopathic pulmonary fibrosis is thought to lead to the presence of more l-ornithine in lung environment [19]. l-ornithine is also known to promote *Pa* biofilm formation in vitro [20]. We hypothesize that l-ornithine serves as a chemoattractant to the opportunistic pathogen *Pa*.

## 2. Materials and Methods

### 2.1. Bacterial Strains and Culture Conditions

The strains used in this study are listed in Table 1 and Supplementary Table S1. PAO1 and the 24 MCP single mutants of PAO1 were obtained from Dr. Junichi Kato (Hiroshima University, Japan). The clinical isolates of *Pa* from acute infections and one environmental strain were kindly provided by Dr. Joseph D. Schwartzman and Dr. Michael Zegans (Geisel School of Medicine, Dartmouth College). Prior to being used in chemotaxis assays, all strains were incubated overnight at 37 °C in minimal salts medium (MSB) [21] supplemented with 0.5% (*w*/*v*) casamino acids (Amresco, Solon, OH, USA) and 27.5 mM glucose.

### 2.2. DNA Manipulation and Electroporation

Standard procedures were followed for the manipulation of plasmid DNA as described previously [28]. To generate PCT2 pMAI18-1(*pctA*), PCT2 pMAI18-1(*pctB*), and PCT2 pMAI18-1(*pctC*), PCT2 was transformed by electroporation [29] with the vector pMAI18-1 (carrying *pctA*), pMAI18-2 (carrying *pctB*), and pMAI18-3 (carrying *pctC*) [27], respectively.

### 2.3. L-Ornithine Purity Analysis

We checked the purity of l-ornithine used for chemotaxis assays by LC/MS/MS analysis (without chemical derivatization) on a Waters Quattro micro-mass spectrometer coupled to Shimadzu high performance liquid chromatography (HPLC), as described previously [30]. The purity analysis of l-ornithine with HPLC revealed that it has low percentage (0.1%) of arginine contamination. Statistical analysis clearly shows that PAO1 chemotaxis response to l-ornithine is not the consequence of arginine contamination (ANOVA test (*p* ≤ 0.05), *p* = 0.034).

### 2.4. Chemotaxis Assays

Three l-ornithine, l-arginine, and casamino acids used for the study were obtained from Amresco (Solon, OH, USA) at the highest purity commercially available. All the test compounds for chemotaxis assays were prepared in chemotaxis buffer (CB) containing 50 mM sodium phosphate buffer (pH 7.0), 10 mM disodium EDTA and 0.05% (*w*/*v*) glycerol.

The qualitative capillary assays were carried out as described previously [31,32]. Briefly, bacterial cells were harvested in mid-exponential phase (OD_660_ 0.3–0.4) by centrifugation at 4600 revolutions per min (rpm) for 5 min and washed once with CB. Washed cells were suspended in CB and diluted to OD_660_ 0.1 and then placed in a chemotaxis chamber formed by a coverslip and a glass U-tube. Microcapillaries (1 µL) were filled with 10 mM of each test compounds in a gel of 2% (*w*/*v*) low-melting-temperature agarose (Nusieve GTG; Lonza, Switzerland) in CB and inserted into the pool of bacterial cells. The amino acids l-ornithine and l-arginine were tested at 0.001, 0.01, 0.1, 1 and 10 mM. In all experiments, negative controls (CB) and positive controls (0.2%, *w*/*v*, casamino acids) were included. The responses were visualized at 0 and 5 min via an Olympus IX73 inverted microscope with an Olympus TH4-100 halogen illuminator and photographed using an Olympus DP73 CCD camera with Olympus cellSens standard version 1.8 software. The dark-field illumination was generated using a Ph2 ring in the long-working distance condenser NA 0.55 with a UPlanFLN 4X NA. 0.13 objective. Images were processed (contrast and brightness) as well as centered using Adobe Photoshop Lightroom. The data were normalized by subtracting the response seen at 0 min from response seen at 5 min using the Matlab R2013a program to obtain a heat map of bacterial chemotactic responses. Two independent replicates and two technical repeats of assays were performed per test samples.

Quantitative capillary assays were carried out as described previously [31,33]. Briefly, bacterial cells were harvested in mid-exponential phase (OD_660_ 0.3–0.4) by centrifugation (4600 rpm) for 5 min and washed once with CB. Cells were then resuspended and diluted to an OD_660_ of 0.1. The capillary tubes consisted of 1μL disposable micropipettes with one end sealed via flame and filled with the chemoattractant resuspended in CB. After incubation in the pool of cells for 30 min at room temperature, the capillary was removed, the exterior was rinsed with sterile chemotaxis buffer, and the contents of the capillaries were transferred to tubes of CB via centrifugation (8000 rpm). Dilution in CB and then plating allowed for determination of the number of colony forming units per capillary (CFUs/capillary). The amino acids l-ornithine and l-arginine were tested at 0.001, 0.01, 0.1, 1 and 10 mM. In all experiments, negative controls (CB) and positive controls (0.2%, *w*/*v*, casamino acids) were used. Data are represented as the mean ± SEM of at least four independent experiments with two technical replicates each. The results have been normalized with CB.

## 3. Results

### 3.1. PAO1 Chemotaxis towards l-ornithine

**Figure 1:**
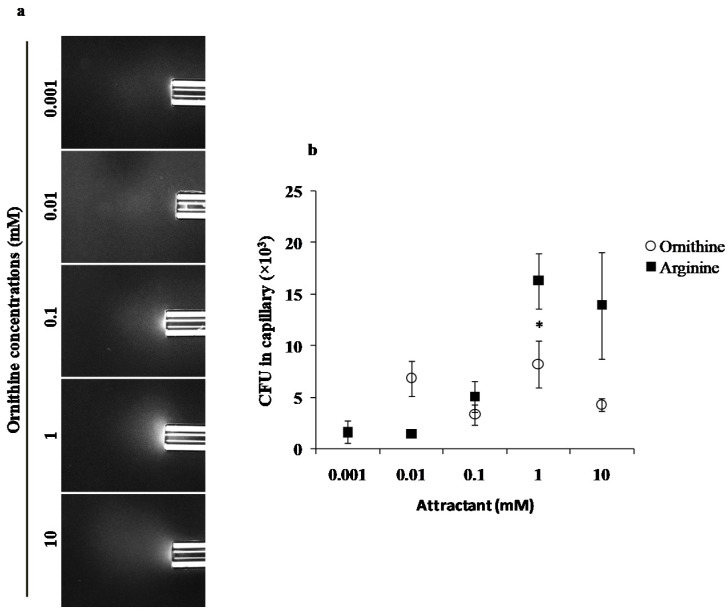
Capillary chemotaxis assays of PAO1 towards different l-ornithine concentrations. (**a**) Dark-field images of cells gathered at the mouth of capillaries containing attractants. All photographs were taken after 5 min. A normalization of the response visualized at 5 min with respect to the time 0 min for each treatment is represented with a jet Colormap (MATLAB R2013b version 8.2) in Supplementary Figure S1. (**b**) Quantitative chemotaxis response of PAO1 towards different l-ornithine and l-arginine concentrations. Results are averages of at least 12 capillaries from five independent experiments; the results have been normalized with CB as the negative control; error bars indicate standard errors, * *p* < 0.05 (by Tukey’s test).

We evaluated the chemotaxis potential of PAO1 and l-ornithine using standard qualitative (Figure 1a) and quantitative (Figure 1b) capillary chemotaxis assays. PAO1 cells formed clearly visible clouds of turbidity because of their accumulation around the open end of capillaries containing 0.1, 1 and 10 mM ornithine (Figure 1a and Supplementary Figure S1). The observations were further confirmed by quantitative assays carried out in the same range of ornithine concentrations (Figure 1b). We observed the maximum chemotaxis response at 1 mM concentration. The chemotaxis response of PAO1 to arginine was included for direct comparison (Figure 1b).

### 3.2. L-Ornithine Chemotaxis Is Mediated by PctA and PctB Receptors in PAO1

**Figure 2:**
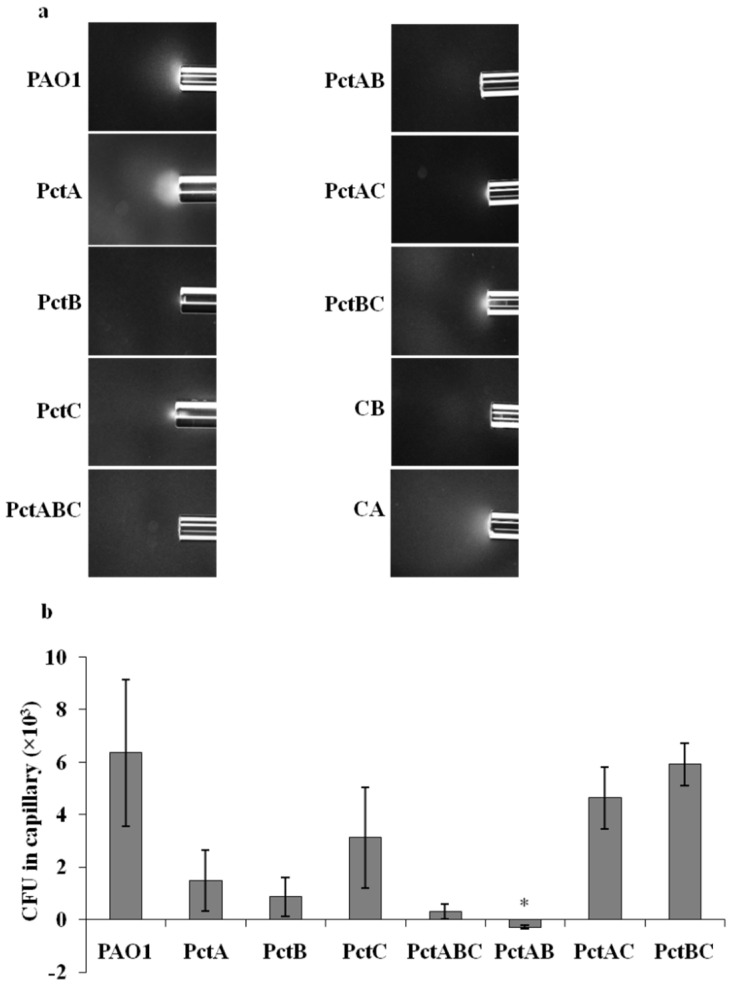
Capillary chemotaxis assays of wild-type PAO1, its mutant PAO1 *ΔpctA* (PctA), PAO1 *ΔpctB* (PctB), PAO1 *ΔpctC* (PctC), PAO1 *ΔpctABC* (PctABC), PAO1 *ΔpctAB* (PctAB), PAO1 *ΔpctAC* (PctAC) and PAO1 *ΔpctBC* (PctBC) towards 1 mM l-ornithine. (**a**) Dark-field images of cells gathered at the mouth of capillaries containing attractants. All photographs were taken after 5 min. A normalization of the response visualized at 5 min with respect to the time 0 min for each treatment is represented with a jet Colormap (MATLAB R2013b version 8.2) in Supplementary Figure S3. Chemotaxis buffer (CB) and casamino acids (CA) were used as negative and positive control, respectively. (**b**) Quantitative comparison of l-ornithine chemotaxis response between the wild-type strain and its mutants indicated by Dunn’s test (* *p* < 0.05). The graph shows CFU in capillaries normalized by the negative control (CB). Results are averages of at least four independent experiments; error bars indicate standard errors.

Chemotaxis receptors PctA, PctB, and PctC mediate chemotaxis towards proteinogenic amino acids, some non-proteinogenic amino acids, and some intermediates of amino acids metabolism for PAO1 [14,15]. L-Ornithine is a non-proteinogenic amino acid derived from metabolism of arginine, thus, we hypothesized that it might serve as an attractant for PAO1, mediated by some or all of these three receptors. In order to identify the chemoreceptors involved in l-ornithine chemotaxis, we screened mutants in the presence of 1mM l-ornithine in which single chemoreceptor genes (*pctA*, *pctB*, *pctC*), and all three chemoreceptor genes (triple mutant with all three *pctA*, *pctB*, and *pctC*) had been deleted (Table 1). The PctABC triple mutant (PCT2) almost completely abolished chemoattraction towards l-ornithine compared to the wild-type strain (Figure 2). The single mutant strains PctA and PctB also showed strongly reduced chemoattraction to l-ornithine (Figure 2), indicating the involvement of both chemoreceptors in l-ornithine chemotaxis for PAO1. The chemoreceptors PctA, PctB, and PctC were reconstituted in the PctABC triple mutant strain to effectively generate double knockout mutants PctBC, PctAC, and PctAB, respectively. The quantitative capillary assays of PctAC and PctBC mutant strains to l-ornithine resulted in significant chemotaxis, whereas PctAB abolished chemotaxis (Figure 2). This confirms that mutation in both PctA and PctB receptors are necessary to completely abolish chemotaxis of PAO1 towards l-ornithine. Additionally, to rule out the involvement of other receptors, we performed qualitative capillary assays with other 21 MCP single mutants (Supplementary Table S1). All MCP mutants showed chemotaxis response to l-ornithine at 1 mM (Supplementary Figure S2), suggesting that these receptors are not involved in l-ornithine chemotaxis.

### 3.3. Diverse Pa Isolates Are Attracted to l-ornithine

**Figure 3:**
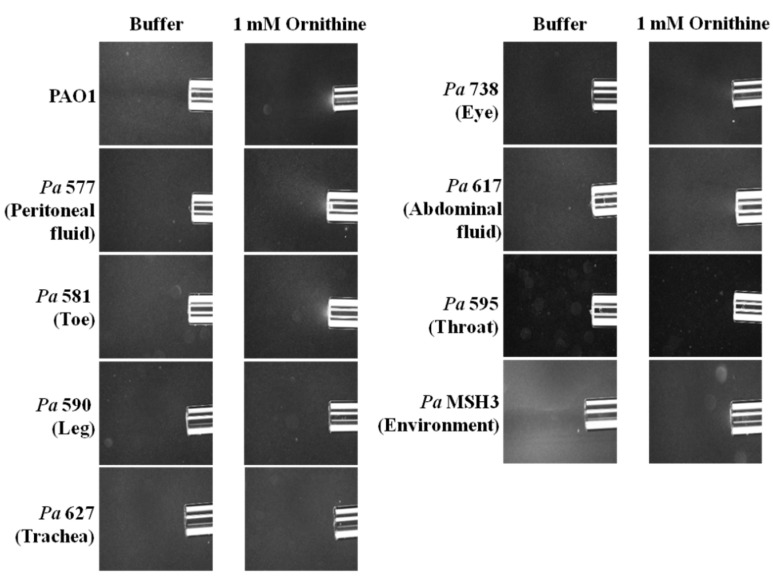
Qualitative capillary chemotaxis assays comparing responses of PAO1 and other *P. aeruginosa* strains (seven clinical isolates and one environmental strain) to 1 mM l-ornithine. Dark-field images of cells gathered at the mouth of capillaries containing attractants. All photographs were taken after 5 min. A normalization of the response visualized at 5 min with respect to the time 0 min for each treatment is represented with a jet Colormap (MATLAB R2013b version 8.2) in Supplementary Figure S4.

To evaluate if this phenomenon of l-ornithine chemotaxis might be generalizable to other *Pa* strains, we conducted qualitative chemotaxis assays on diverse eight, motile *Pa* strains (seven clinical isolates and one environmental strain) (Table 1). These clinical strains were isolated from patients with acute peritoneal fluid, toe, leg, trachea, eye, abdominal fluid and throat infections. Three strains, originating from peritoneal fluid, toe and abdominal fluid infections, exhibited putative chemotaxis to l-ornithine (Figure 3).

## 4. Discussion

The chemoreceptors PctA, PctB, and PctC are known to mediate PAO1 chemotaxis towards 20 naturally occurring amino acids [14,23]. PctA, PctB and PctC have individual specificities for L-amino acids in spite of their highly similar periplasmic domain. PctA detects 18 out of 20 naturally occurring L-amino acids, while PctB and PctC respond to seven and two naturally occurring L-amino acids, respectively [14]. PctC also responds to the non-proteinogenic amino acid gamma-aminobutyric acid [15]. Plants and animals have several other chemically diverse, reasonably abundant, and physiologically important non-proteinogenic amino acids and intermediates of amino acid metabolism, which are good candidates for chemotaxis evaluation, especially in the context of potential influences on bacterial pathogenesis.

Herein, we demonstrated that PAO1 is attracted to l-ornithine in the concentration range of 0.01 to 10 mM (Figure 1), which is similar to other amino acids [14,34], and which is specifically slightly lower in response compared to arginine (Tukey’s honest significant difference test (*p* ≤ 0.05) at concentrations of 10 mM, *p* = 0.003). Previous studies have shown that paralogous chemoreceptors of a particular bacterial strain can mediate chemotaxis to the same ligand. For instance, *P. putida* KT2440 strains, McpS and McpQ chemoreceptors, respond to citrate [35,36], and PctA and PctB of *Pa* for proteinogenic amino acids [14,15]. Here, we found that paralogous receptors PctA and PctB are responsible for the chemoattraction response of PAO1 to l-ornithine and possibly, by extension, to the other *Pa* isolates showing chemoattraction evaluated in this study. Arginine, a precursor proteinogenic amino acid for l-ornithine production, also uses the PctA and PctB receptors [14].

Previous studies suggest that Pct chemoreceptors are associated with *Pa* virulence. For example, PctB-mediated chemotaxis to glutamine [14,15], the most abundant amino acid in human cells [37], potentially relates to virulence, and this receptor might be especially valuable for efficient host colonization by *Pa* [38]. In addition, a PctABC triple mutant was reported to be less efficient in colonizing wounds of human CF airway epithelial cells [39]. Furthermore, PctA and PctB protein levels are downregulated in *Pa* isolated from the sputum of patients with CF [40]. Therefore, taken together, our work adds to the growing suggestion that amino acid chemotaxis is likely primarily important for the initial stages of bacterial attachment and infection in humans and animals, where amino acid concentrations, including l-ornithine [41], in sputum or lung are high, especially in patients with CF [42]. Further studies are required to evaluate precise and specific roles of amino acid chemotaxis on the fate and activity of *Pa* in human and animal infections.

## Figures and Tables

**Table 1:** Bacterial Strains and plasmids.

Strain or Plasmid	Characteristics	Reference or Source
Strains		
*Pseudomona aeruginosa* PAO1	Prototroph, FP (sex factor minus)	[22]
PCT2	PAO1 derivative, Δ*pctC*, Δ*pctA*, Δ*orfl*, Δ*pctB*::Km	[14]
PCTA1	PAO1 derivative, Δ*pctA*::Km^r^	[23]
PCTB1	PAO1 derivative, Δ*pctB*::Km^r^	[14]
PCTC1	PAO1 derivative, Δ*pctC*::Km^r^	[14]
PCT2pMAI18-1(*pctA*)	PAO1 derivative, Δ*pctB,* Δ*pctC*::Cb^r^	This study
PCT2pMAI18-1(*pctB*)	PAO1 derivative, Δ*pctA,* Δ*pctC*::Cb^r^	This study
PCT2pMAI18-1(*pctC*)	PAO1 derivative, Δ*pctA,* Δ*pctB*::Cb^r^	This study
*P. aeruginosa* 577	Clinical isolate from peritoneal fluid	PA8 ^§^
*P. aeruginosa* 581	Clinical isolate from toe	PA1 ^§^
*P. aeruginosa* 590	Clinical isolate from leg	PA2 ^§^
*P. aeruginosa* 627	Clinical isolate from trachea	PA42 ^§^
*P. aeruginosa* 738	Clinical isolate from eye	[24]
*P. aeruginosa* 595	Clinical isolate from throat	PA6 ^§^
*P. aeruginosa* 617	Clinical isolate from abdominal fluid	PA36 ^§^
*P. aeruginosa* MSH3	Environmental strain	[25]
Plasmids		
pUCP18	Broad-host-range cloning vector; Cb^r^	[26]
pMAI18-1	pUCP18 with *pctA*(2.1 kb), Cb^r^	[27]
pMAI18-2	pUCP18 with *pctB*2.1 kb); Cb^r^	[27]
pMAI18-3	pUCP18 with *pctC*(2.1 kb), Cb^r^	[27]

Km, kanamycin; Cb, carbenicillin. ^§^ Culture collections obtained from Dr. Schwartzman (Geisel School of Medicine, Dartmouth).

## Data Availability

Data are presented in the paper and the supporting information. Original data files will be available upon request from the authors.

## Supplementary Materials

The following are available online at https://www.mdpi.com/article/10.3390/microorganisms10020264/s1; Figure S1. Normalized jet Colormap of qualitative capillary chemotaxis responses of wild-type PAO1 towards different concentrations of ornithine (mM). A normalization of the response visualized at 5 min respect to the time 0 min for each treatment is represented with a jet Colormap (MATLAB R2013b version 8.2). Figure S2. Qualitative capillary chemotaxis assays of 21 MCP mutants of PAO1 towards ornithine (1 mM). Dark-field images of cells gathered at the mouth of capillaries containing ornithine (first, third and fifth columns). All photographs were taken after 5 min. A normalization of the response visualized at 5 min with respect to the time 0 min for each treatment is represented with a jet Colormap (MATLAB R2013b version 8.2) (second, fourth and sixth column). Figure S3. Normalized jet Colormap of qualitative capillary chemotaxis responses of wild-type PAO1, its mutant *P. aeruginosa* PAO1 *ΔpctA* (PctA), PAO1 *ΔpctB* (PctB), PAO1 *ΔpctC* (PctC), PAO1 *ΔpctABC* (PctABC), PAO1 *ΔpctAB* (PctAB), PAO1 *ΔpctAC* (PctAC) and PAO1 *ΔpctBC* (PctBC) towards 1 mM l-ornithine. A normalization of the response visualized at 5 min with respect to the time 0 min for each treatment is representing with a jet Colormap (MATLAB R2013b version 8.2). Chemotaxis buffer (CB) and casamino acids (CA) were used as negative and positive control, respectively. Figure S4. Normalized jet Colormap of qualitative capillary chemotaxis responses of PAO1 and other *Pa* strains (seven clinical isolates and one environmental strain) to 1 mM l-ornithine. A normalization of the response visualized at 5 min with respect to the time 0 min for each treatment is represented with a jet Colormap (MATLAB R2013b version 8.2). Table S1. *P. aeruginosa* PAO1 strain and MCP single mutants of PAO1.
