# Supplementary material for: The Arginine Catabolism-Derived Amino Acid l-ornithine Is a Chemoattractant for Pseudomonas aeruginosa
Source: Microorganisms. 2022 Jan 24;10(2):264. doi: 10.3390/microorganisms10020264 (PMC8875649; doi:10.3390/microorganisms10020264)
Supplement: Supplementary file 1 [file microorganisms-10-00264-s001.zip › microorganisms-1510018-supplementary.pdf]

Supplementary Materials

The Arginine Catabolism-Derived Amino Acid L-ornithine Is a Chemoattractant for *Pseudomonas aeruginosa*

Basanta Dhodary[1,*], Inmaculada Sampedro[2,3,4,*], Shekooh Behroozian[1], Victor Borza[2], Stephanie Her[2], Jane E. Hill[1,2, †]

[1]*School of Biomedical Engineering, University of British Columbia, Vancouver, British Columbia, Canada*

[2]*Thayer School of Engineering, Dartmouth College, Hanover, NH, US*

[3]*Biomedical Research Center (CIBM), Biotechnology Institute, Avda del Conocimiento s/n, 18100 Armilla, Spain*

[4]*Department of Microbiology, Faculty of Pharmacy, University of Granada, Campus de Cartuja s/n, 18071 Granada, Spain*

[†]Corresponding author

*These authors contributed equally.

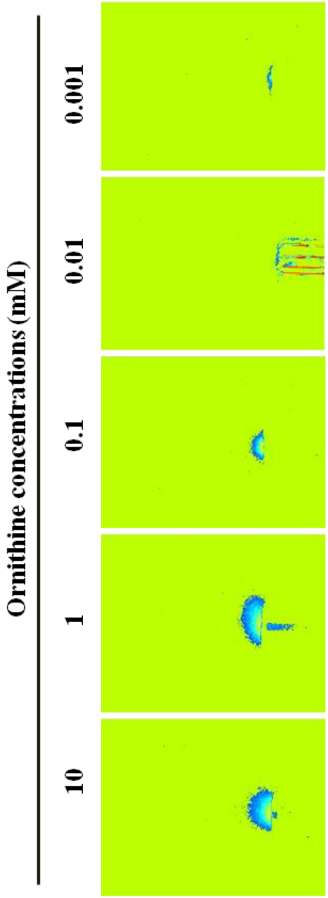

Figure S1: Normalized jet Colormap of qualitative capillary chemotaxis responses of wild-type PAO1 towards different concentrations of ornithine (mM). A normalization of the response visualized at 5 min respect to the time 0 min for each treatment is representing with a jet Colormap (MATLAB R2013b version 8.2).

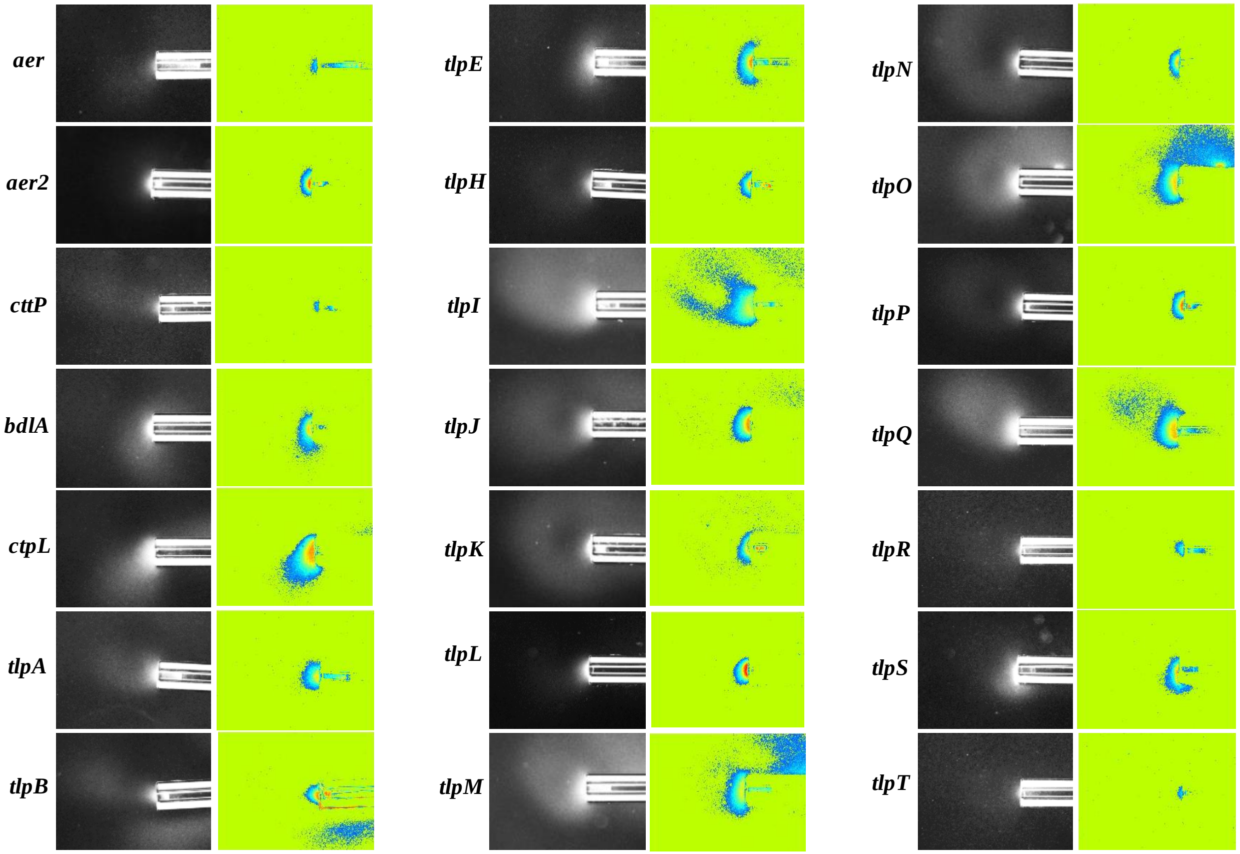

Figure S2: Qualitative capillary chemotaxis assays of 21 MCP mutants of PAO1 towards ornithine (1 mM). Dark-field images of cells gathered at the mouth of capillaries containing ornithine (first, third and fifth columns). All photographs are taken after 5 min. A normalization of the response visualized at 5 min with respect to the time 0 min for each treatment is representing with a jet Colormap (MATLAB R2013b version 8.2) (second, fourth and sixth column).

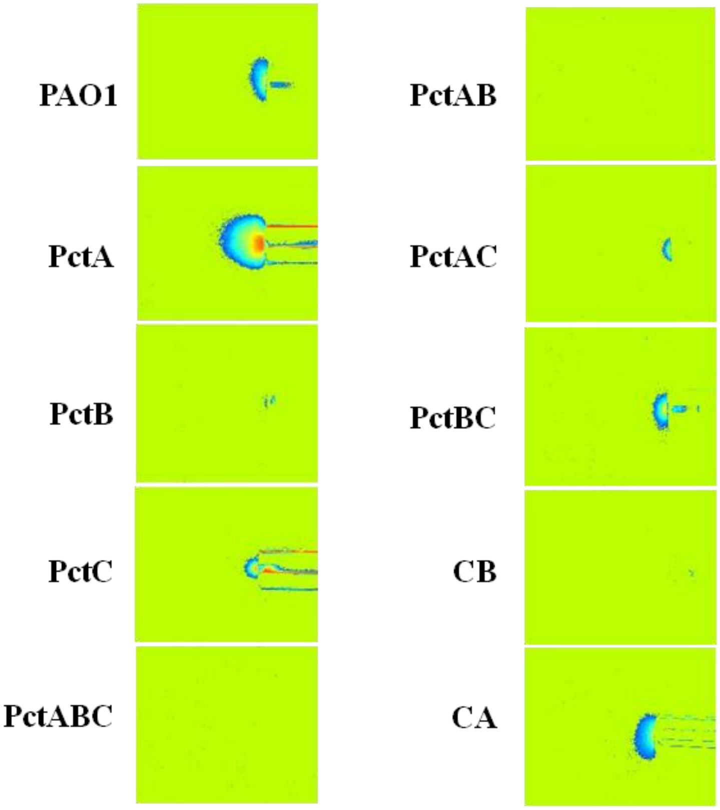

Figure S3: Normalized jet Colormap of qualitative capillary chemotaxis responses of wild-type PAO1, its mutant *P. aeruginosa* PAO1 *ΔpctA* (PctA), PAO1 *ΔpctB* (PctB), PAO1 *ΔpctC* (PctC), PAO1 *ΔpctABC* (PctABC), PAO1 *ΔpctAB* (PctAB), PAO1 *ΔpctAC* (PctAC) and PAO1 *ΔpctBC* (PctBC) towards 1 mM L-ornithine. A normalization of the response visualized at 5 min with respect to the time 0 min for each treatment is representing with a jet Colormap (MATLAB R2013b version 8.2). Chemotaxis buffer (CB) and Casamino acids (CA) were used as negative and positive control, respectively.

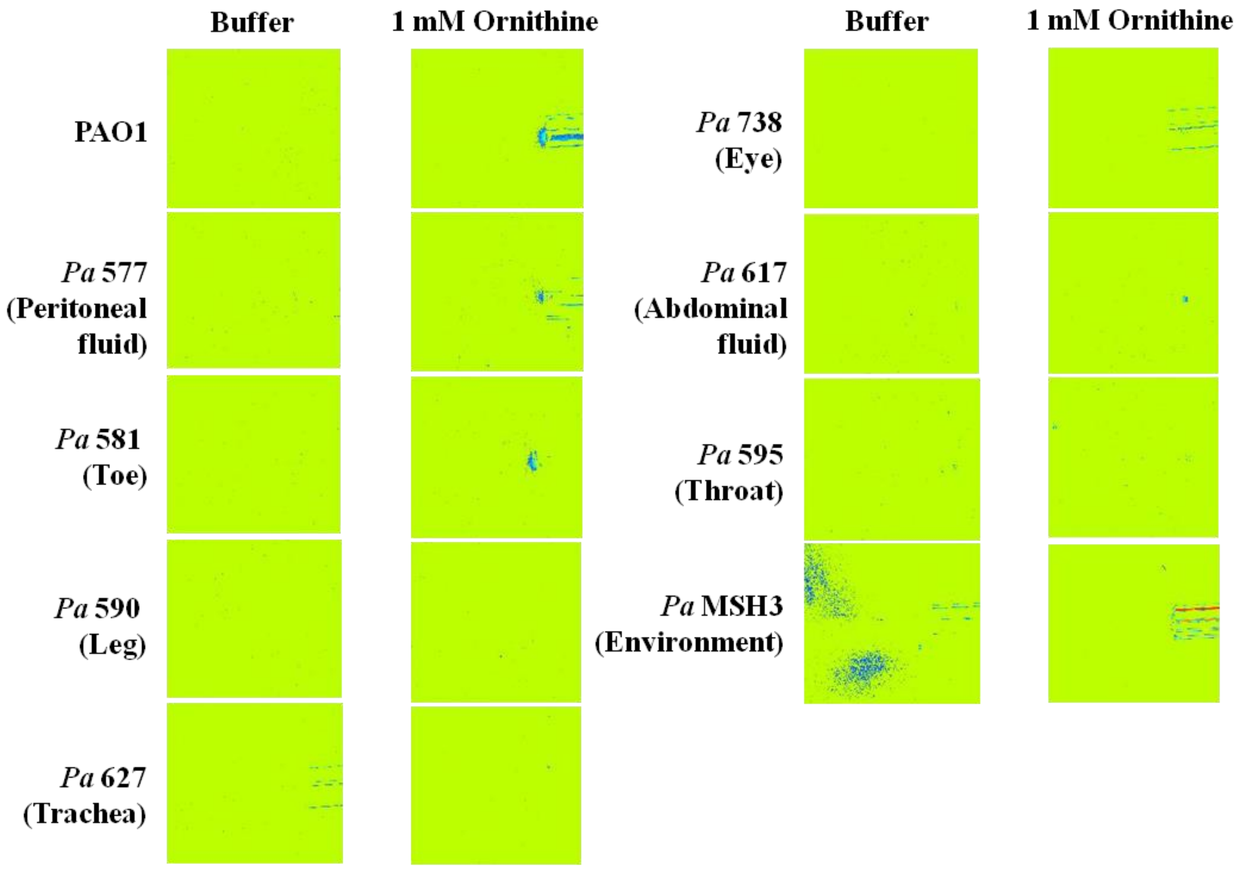

Figure S4: Normalized jet Colormap of qualitative capillary chemotaxis responses of PAO1 and other *Pa* strains (seven clinical isolates and one environmental strain) to 1 mM L-ornithine. A normalization of the response visualized at 5 min with respect to the time 0 min for each treatment is representing with a jet Colormap (MATLAB R2013b version 8.2).

Table S1: *P. aeruginosa* PAO1 strain and MCP single mutants of PAO1

**Strains**	**Characteristics**	**Reference or source**
Pseudomona aeruginosa PAO1	Prototroph, FP (sex factor minus)	[1]
tlpA	PAO1 derivative, Δ*tlpA*::Km^r	PA1646[*]
tlpB	PAO1 derivative, Δ*tlpB*::Km^r	PA1608[*]
tlpE	PAO1 derivative,Δ*tlpE*::Km^r	PA1251[*]
tlpH	PAO1 derivative, Δ*tlpH*::Km^r	PA5072[*]
tlpI	PAO1 derivative, Δ*tlpI*::Km^r	PA4915[*]
tlpJ	PAO1 derivative, Δ*tlpJ*::Km^r	PA4633[*]
tlpK	PAO1 derivative, Δ*tlpK*::Km^r	PA4520[*]
tlpL	PAO1 derivative, Δ*tlpL*::Km^r	PA4290[*]
wspA (*tlpM*)	PAO1 derivative,Δ*tlpM*::Km^r	PA3708[*]
tlpN	PAO1 derivative, Δ*tlpN*::Km^r	PA2920[*]
tlpO	PAO1 derivative, Δ*tlpO*::Km^r	PA2867[*]
tlpP	PAO1 derivative, Δ*tlpP*::Km^r	PA2788[*]
tlpQ	PAO1 derivative, Δ*tlpQ*::Km^r	[2]
mcps (*tlpR*)	PAO1 derivative, Δ*tlpR*::Km^r	[3]
tlpS	PAO1 derivative, Δ*tlpS*::Km^r	PA2573[*]
tlpT	PAO1 derivative, Δ*tlpT*::Km^r	PA1930[*]
cttP	PAO1 derivative, Δ*cttP*::Km^r	[4]
ctpL	PAO1 derivative, Δ*ctpL*::Km^r	[5]
bdlA	PAO1 derivative, Δ*bdlA*::Km^r	[6]
aer (*tlpC*)	PAO1 derivative, Δ*tlpC*::Km^r	[7]
aer2 (*tlpG*)	PAO1 derivative, Δ*tlpG*::Km^r	[7]

Km, kanamycin. [*]Culture collections obtained from Dr. Kato (Hiroshima University, Japan).

References

1. Holloway, B.; Krishnapillai, V.; Morgan, A. Chromosomal genetics of Pseudomonas. *Microbiol. Rev.* 1979, *43(1)*, 73-102.
2. Kim, H.E.; Shitashiro, M.; Kuroda, A.; Takiguchi, N.; Kato, J. Ethylene chemotaxis in *Pseudomonas aeruginosa* and other Pseudomonas species. *Microbes Environ.* **2007**, *22(2)*, 186-189, doi:10.1264/jsme2.22.186
3. Alvarez-Ortega, C.; Harwood, C.S. Identification of a malate chemoreceptor in *Pseudomonas aeruginosa* by screening for chemotaxis defects in an energy taxis-deficient mutant. *Appl. Environ. Microbiol.* **2007**, *73(23)*, 7793-7795, doi:10.1128/AEM.01898-07
4. Kim, H.E.; Shitashiro, M.; Kuroda, A.; Takiguchi, N.; Ohtake, H.; Kato, J. Identification and characterization of the chemotactic transducer in *Pseudomonas aeruginosa* PAO1 for positive chemotaxis to trichloroethylene. *J. Bacteriol.* **2006**, *188(18)*, 6700-6702, doi:10.1128/JB.00584-06
5. Wu, H.; Kato, J; Kuroda, A.; Ikeda, T.; Takiguchi, N.; Ohtake, H. Identification and characterization of two chemotactic transducers for inorganic phosphate in *Pseudomonas aeruginosa*. *J. Bacteriol.* **2000**, *182(12)*, 3400-3404, doi:10.1128/jb.182.12.3400-3404.2000
6. Sampedro, I.; Kato, J.; Hill, J.E. Elastin degradation product isodesmosine is a chemoattractant for *Pseudomonas aeruginosa*. *Microbiology* **2015**, *161(7)*, 1496-1503, doi:10.1099/mic.0.000090
7. Hong, C.S.; Shitashiro, M.; Kuroda, A.; Ikeda, T.; Takiguchi, N.; Ohtake, H.; Kato, J. Chemotaxis proteins and transducers for aerotaxis in *Pseudomonas aeruginosa*. *FEMS Microbiol. Lett.* **2004**, *231(2)*, 247-252, doi:10.1016/S0378-1097(04)00009-6
